# Glomerular Galactose-Deficient IgA1(KM55) Positive May Predict Poorer Prognosis in Coexisting Primary Membranous Nephropathy and IgA Nephropathy Patients

**DOI:** 10.3390/cells12010116

**Published:** 2022-12-28

**Authors:** Wenrong Cheng, Guoqin Wang, Weiyi Guo, Lijun Sun, Xiaoyi Xu, Hongrui Dong, Suhua Ye, Yanqiu Geng, Hong Cheng

**Affiliations:** 1 Renal Division, Department of Medicine, Beijing Anzhen Hospital, Capital Medical University, Beijing 100029, China; 2 Division of Nephrology, Affiliated Hospital of Chifeng University, Neimenggu 024000, China; 3 Division of Nephrology, The Third Medical Center of Chinese PLA General Hospital, Beijing 100039, China

**Keywords:** galactose-deficient IgA1, KM55, primary membranous nephropathy, IgA nephropathy, IgA deposition, prognosis

## Abstract

Primary membrane nephropathy (PMN) and IgA nephropathy (IgAN) are the most common glomerular diseases in China. Because of different pathogenesis, prognosis is significantly different. When the two diseases coexist (PMN/IgAN), the clinicopathological manifestations and prognosis remain unclear. In the present study, we analyzed the clinicopathological characteristics of PMN/IgAN patients, with only IgA deposition (PMN/IgA deposition) patients as controls. Galactose-deficient IgA1(KM55) and M-type Phospholipase A2 Receptor(PLA2R), both in circulation and renal tissues, were detected. Furthermore, prognosis of PMN/IgAN was explored. We found that PMN/IgAN also had some clinical features of IgAN in addition to PMN, such as higher serum albumin, along with a similar heavy proteinuria and lower titers of serum anti-PLA2R antibody. The positive rate of glomerular KM55 in PMN/IgAN was 23.5% (20/85), and 0% (0/29) in PMN/IgA deposition. Among those glomerular KM55 positive patients, KM55 and IgA colocalized mainly along the glomerular mesangial and capillary areas. Unfortunately, there was no significant difference in serum level of Gd-IgA1 between KM55+ and KM55− subgroups in PMN/IgAN patients, similar to the PMN/IgA deposition group. Notably, glomerular KM55 positive may predict a poorer prognosis in PMN/IgAN patients. In conclusion, our study suggested that, when glomerular KM55 staining was positive, this special coexisting PMN/IgAN disorder was prone to have more characteristics of IgAN besides PMN, and may predict poorer prognosis, while the mechanism requires further investigation.

## 1. Introduction

Primary membranous nephropathy (PMN) and IgA nephropathy(IgAN) are the most common forms of primary glomerular disease (PGD), while PGD remains the leading cause of chronic kidney disease (CKD) and renal failure in patients receiving renal biopsies in China [1,2]. There are significant differences between these two diseases, such as the age of onset, clinical manifestations, pathogenesis, treatment and prognosis. PMN, a common cause of nephrotic syndrome in adults, especially in no-diabetic adults, is characterized by accumulation of immune deposits in the subepithelial space of glomerular capillaries [3]. IgAN displays proteinuria and microscopic or gross hematuria, especially heavy proteinuria, while serum albumin is still not significantly reduced, characterized by the deposition of immune complexes composed of galactose-deficient IgA1 (Gd-IgA1) in the mesangium of glomeruli [4]. The prognoses of these two diseases are significantly different based on distinct pathogenesis. PMN seems to be a benign disease, whose main target antigen is M-type Phospholipase A2 Receptor(PLA2R); about one third of patients show spontaneous remission and only 10% or fewer will develop end-stage renal disease(ESRD) over the subsequent 10 years with proper management [3,5]. Meanwhile, about 20–40% of IgAN patients are associated with accelerated disease progression and progress to ESRD within 10 years after diagnosis [1].

PMN and IgAN are very common diseases independently and coexisting PMN and IgAN(PMN/IgAN) in the same patient is rare. However, it is not unusual to observe simultaneous IgA deposit in the pathological context of PMN. It is unknown whether this is classic IgAN or just incidental IgA deposition accompanying PMN, and whether this coexisting disorder represents different prognosis. With the intensive study of the pathogenic mechanism of PMN and IgAN, more and more evidence has indicated that PLA2R and Gd-IgA1 play important roles in PMN and IgAN separately. However, the clinicopathological characteristics and pathogenesis of these PMN/IgAN patients have been investigated only by single case or in case series [6,7]. The role of glomerular and serum Gd-IgA1 in these PMN/IgAN patients is rarely discussed. To our knowledge, study of the immunopathogenesis and prognosis of this uncommon overlap of PMN and IgAN is limited.

In this study, we examined Gd-IgA1 and PLA2R simultaneously both in circulation and renal tissues in these PMN/IgAN patients to better understand the pathogenesis, and in addition to evaluate the prognosis in these patients.

## 2. Materials and Methods

### 2.1. The Process to Select Patients with PMN Accompanied IgA Deposition

**Figure 1:**
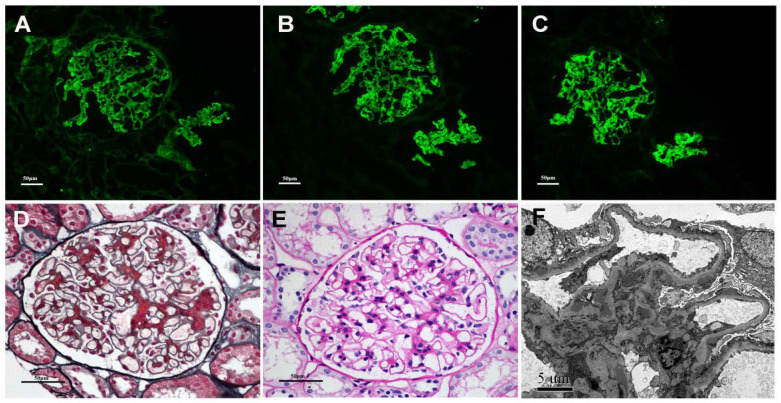
Renal biopsy findings for a PMN/IgAN patient. Immunofluorescence showing IgG (**A**) and IgG4 (**B**) in granular pattern. Immunofluorescence showing IgA (**C**) deposition in mesangial along with capillary area. Light microscopy showing glomerular basement membrane thickening and fuchsinophilic proteins in mesangial region (**D**,**E**). Electron micrograph showing subepithelial and mesangial electron-dense (**F**). (**A**–**C**: Original magnification ×200; **D**,**E**: Original magnification ×400; **F**: Original magnification ×5000).

**Figure 2:**
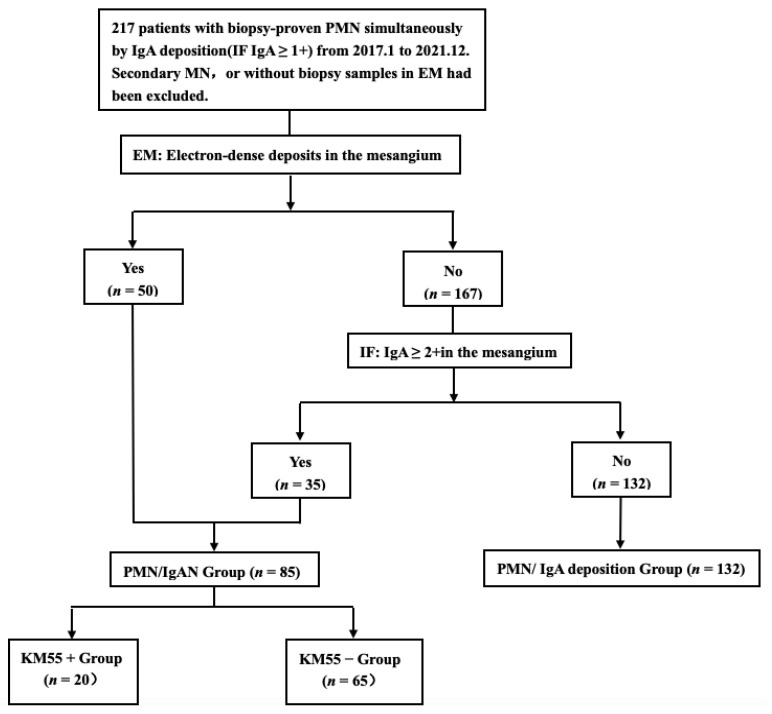
Flowchart of the recruitment process.

A total of 217 patients with biopsy-proven PMN and simultaneous IgA deposition(IgA ≥ 1+) according to immunofluorescence(IF) examination between January 2017 to December 2021 at our center were reviewed retrospectively. Patients with secondary causes, such as autoimmune diseases (e.g., lupus), hepatitis virus infection (hepatitis B or hepatitis C), and toxicants (e.g., mercury), detected at onset or during follow-up, or without biopsy samples via electron microscope(EM), were excluded from our study. The diagnosis of PMN/IgAN was included: (1) with the presence of dominant IgA deposition in the glomerular mesangium on IF and electron-dense deposits in the mesangium on EM (*n* = 50) and (2) IgA was strongly positive (IgA ≥ 2+) in the mesangium, while electron-dense deposits in the mesangium were not observed on EM (*n* = 35) (Figure 1). The rest were considered as controls (PMN/IgA deposition). The flowchart is displayed in Figure 2.

The study was approved by the Ethics Review Committee of Beijing Anzhen Hospital, Capital Medical University, and implemented in accordance with the Declaration of Helsinki. Informed consent was obtained from all enrolled individuals.

### 2.2. Demographics and Clinical Information

Demographic and clinical features at the time of renal biopsy performance included age, gender, co-morbidity, urinary sediment microscopy, 24-h proteinuria excretion, serum levels of IgA, IgG, IgM, C3, C4, uric acid, triglyceride, cholesterol and creatinine. The estimated glomerular filtration rate (eGFR) was calculated by eGFR-EPI formula [8]. Microscopic hematuria was defined as RBC > 3/HP, with microscopic examination of sediment after centrifugation. Hypertension was defined as systolic blood pressure(SBP) exceeding 140 mmHg and/or diastolic blood pressure(DBP) exceeding 90 mmHg, or receiving antihypertensive drugs.

### 2.3. Histological Manifestations

All the kidney sections were processed routinely for light microscopy, direct IF and EM according to the standard procedure [9]. MN was divided into four stages [10]. If two stages were noted at the same time, the relatively higher stage was selected. The severity of interstitial fibrosis was recorded according to the percentage of the affected area of the interstitium as <25.0%, 25.0–50.0%, 50.0–75.0%, >75.0%.

### 2.4. Outcomes

The definitions of remission complied with the 2012 Kidney Disease Improving Global Outcomes guideline for glomerular nephropathy [11]. Complete remission was defined as urinary protein excretion <0.3 g/d, confirmed by two values at least 1 week apart, accompanied by normal serum albumin and creatinine levels. Partial remission was defined as urinary protein excretion <3.5 g/d and at least a 50% reduction from peak values accompanied by an improvement or normalization of serum albumin and stable serum creatinine levels. The composite endpoint in this study was defined as a 25% decline in eGFR or ESRD, whichever occurred first.

### 2.5. Detection of Glomerular Gd-IgA1

Glomerular Gd-IgA1(KM55) deposition was detected by IF. Paraffin-embedded sections (4 μm) of formalin-fixed kidney tissues were deparaffinized and hydrated. Antigen retrieval using pepsin was performed at 37 °C for 35 min. The sections were rinsed with PBS, followed by incubation with 3% bovine serum albumin (BSA, Sigma Chemical Company, St. Louis, MO, USA) for 30 min at room temperature. Primary antibodies (KM55, Immuno-Biological Laboratories, Fujioka, Japan) with a dilution of 1:200 in PBS were incubated overnight at 4 °C. After 3 washes with PBS, CyTM3-conjugated affinipure donkey anti-rat IgG antibody (diluted in 1:200; Jackson ImmunoResearch Laboratories, Philadelphia, PA, USA) was added to the sections at 37 °C for 60 min. The sections were then washed with PBS 3 times and the sections were air-dried in the dark and mounted with mounting medium with DAPI.

The method to detect colocalization of KM55 and IgA (FITC-conjugated rabbit anti-human IgA, Dako, Glostrup, Denmark) were as previously described [12]. As blank controls, primary antibodies were replaced by PBS.

The IF was scored using a fluorescence microscope (Nikon 80i, Japan). Two observers (Wen-rong Cheng and Wei-yi Guo), who were blinded to the clinical data, graded the staining intensity from anonymized sections as follows: negative (0), mild (1+), moderate (2+), strong (3+) [12]. Staining intensity 1+, 2+, 3+ were described as “positive”, while “negative” included 0. Sections that contained fewer than 2 glomeruli were excluded.

To analyze the fluorescence colocalization, two-dimensional (2D) fluorograms were plotted from red and green images by Image Pro Plus software. A 2D fluorogram is a dot diagram that visualizes the joint distribution of intensity values of two detection channels (red and green). Each dot of the scatter plot represents an intensity value pair from the two detection channels [12].

### 2.6. Measurement of Serum Gd-IgA1

Serum samples of 38 patients in the PMN/IgAN group and 19 patients in the PMN/IgA deposition group were collected on the day of biopsy and stored at −80 °C. Levels of serum Gd-IgA1 were measured using a commercially available enzyme-linked immunosorbent assay test kit with KM55 (Immuno-Biological Laboratories, Fujioka, Japan) according to the manufacture’s protocol.

### 2.7. Detection of Glomerular PLA2R, Thrombospondin Type 1 Domain-Containing 7A(THSD7A), and Neural Epidermal Growth Factor–Like 1(NELL-1) Expression and Circulating Anti-PLA2R Antibody

Immunohistochemistry staining for PLA2R, THSD7A and NELL-1 was performed as previously described [9,13,14]. The rabbit anti-human PLA2R polyclonal antibody (1:800; Sigma-Aldrich), the rabbit anti-human THSD7A polyclonal antibody (1:1500; Sigma Chemical Company and the rabbit polyclonal anti–NELL-1 antibody (1:800; Sigma Chemical Company) were the primary antibodies. The serum anti-PLA2R antibody levels were detected by ELISA (EUROIMMUN, Lübeck, Germany). The results were considered positive at ≥ 20 relative units (RU)/mL.

### 2.8. Statistical Analysis

SPSS 25.0 statistical software was utilized for data analysis. Quantitative variables with normal distributions were expressed as x ± s and compared by *t*-tests or one way ANOVA and data with abnormal distributions were expressed as median and interquartile range (IQR), compared by nonparametric test. The qualitative variables were compared by the χ2 test or Fisher exact tests. A poor event-free renal survival curve was prepared using the Kaplan-Meier method by log-rank test. *p* value < 0.05 was considered statistically significant. 

## 3. Results

### 3.1. Baseline Demographic and Clinical Characteristics of PMN/IgAN by Traditional Diagnostic Criteria

A total of 217 patients were enrolled in this study, of which 85 met the traditional diagnostic criteria of PMN/IgAN, accounting for 39.2% of PMN simultaneous IgA deposition. Demographics and clinical features of patients are summarized in Table 1. Compared to PMN/IgA deposition group, patients with PMN/IgAN had higher serum albumin (27.4 versus 25.9 g/L, *p* = 0.049), but a similar 24-h proteinuria excretion. They had a lower frequency of nephrotic syndrome (62.4% versus 77.3%, *p* = 0.018). The level of serum IgG was higher (6.7 versus 5.7 g/L, *p* = 0.004) and serum IgA was higher (2.5 versus 2.1 g/L, *p* = 0.015). The gender distribution, age of onset, kidney function, lever of serum C3, C4, and microscopic hematuria frequency were comparable in the two groups (*p* > 0.05).

### 3.2. Pathological Findings of PMN/IgAN by Traditional Diagnostic Criteria

All the enrolled patients presented with Churg stage I and II of PMN, but the proportion of stage II was lower in the PMN/IgAN group (43.5% versus 62.9%, *p* = 0.019). The PMN/IgAN patients were accompanied by more glomerular IgM deposition(29.4% versus 15.2%, *p* = 0.011). There was no significant difference in the distribution of positive glomerular IgG, IgG subclass, predominant IgG4, C1q, C3, or degree of interstitial fibrosis. Details of the pathological features are shown in Table 2.

### 3.3. Disease-Specific Pathogenic Biomarkers of PMN/IgAN

#### 3.3.1. Glomerular KM55 Staining and Colocalization of KM55 and IgA on Glomeruli in Patients with PMN/IgAN

**Figure 3:**
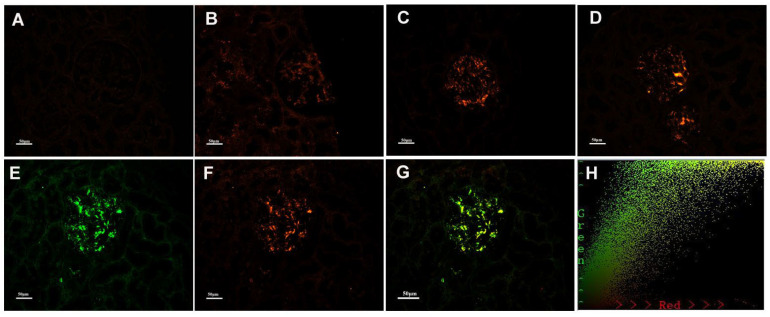
Representative images of immunofluorescence staining for KM55 in patients with PMN/IgAN, (**A**) Negative, (**B**) 1+ intensity, (**C**) 2+ intensity, (**D**) 3+ intensity. Positive staining of KM55 3+ (**F**) by immunofluorescence along the glomerular mesangial and capillary area in the same section with IgA positive staining (**E**). (**G**) KM55 and IgA colocalized completely along the glomerular mesangial and capillary area. The corresponding two-dimensional (2D) fluorograms have been included to confirm the degree of co-localization (**H**: Pearson’s correlation 0.9182, Overlap coefficient 0.9245;). (**A**–**G**: original magnification ×200).

KM55 was deposited mainly in the mesangial and occasionally capillary areas in 20/85 (23.5%) patients with PMN/IgAN, but in 0% (0/29) of patients randomly selected from the PMN/IgA deposition group. Granular positive staining of KM55 by IF occurred along the glomerular mesangial and capillary area in patients with PMN/IgAN (Figure 3). The intensity of glomerular KM55 in the PMN/IgAN group exhibited 0/1+/2+/3+: 65 (76.5%)/8 (9.4%)/10 (11.8%)/2 (2.4%), Table 3 and Figure 3).

As 20/85 (23.5%) patients in the PMN/IgAN group presented with granular positive staining of KM55 along the glomerular mesangial and capillary areas, we further investigated the colocalization of KM55 and IgA. KM55 and IgA along the glomerular mesangial and capillary areas (Figure 3). To quantify the colocalization, two-dimensional (2D) fluorograms have been included to confirm the degree of co-localization (Figure 3H: Pearson’s correlation 0.9182, Overlap coefficient 0.9245).

#### 3.3.2. Serum and Glomerular PLA2R

Patients with PMN/IgAN had a lower frequency of serum anti-PLA2R antibody positivity (43.5% versus 61.4%, *p* = 0.010; 20 RU/mL as the cut-off value) and lower titers of antibody (8.2 versus 47.1 RU/mL, *p* < 0.001). The positive rates of tissue staining for PLA2R were lower in PMN/IgAN, compared to PMN/IgA deposition group (76.5% versus 98.5%, *p* < 0.001).

### 3.4. Baseline Clinical and Pathological Characteristics between Glomerular KM55+ Subgroup and KM55− Subgroup in PMN/IgAN Patients

Compared to patients in KM55− subgroup, patients in KM55+ subgroup had a higher frequency of hypertension (60% versus 32.3%, *p* = 0.026) and a lower level of 24-h proteinuria excretion (3.8 versus 5.8 g/d, *p* = 0.040). All the other demographic features and serum test results were comparable in the two groups (Table 4). Additionally, the proportion of Churg stage I was lower, while Churg stage II was higher in the KM55+ group (*p* = 0.017). Patients in KM55+ subgroup had severer interstitial fibrosis. There was no significant difference in the distribution of positive glomerular IgG, IgG subclass and predominant IgG subclass (IgG4), C1q, C3. Details of the pathological features are shown in Table 5.

### 3.5. Disease-Specific Pathogenic Biomarkers between Glomerular KM55+ Subgroup and KM55− Subgroup in PMN/IgAN Patients

**Figure 4:**
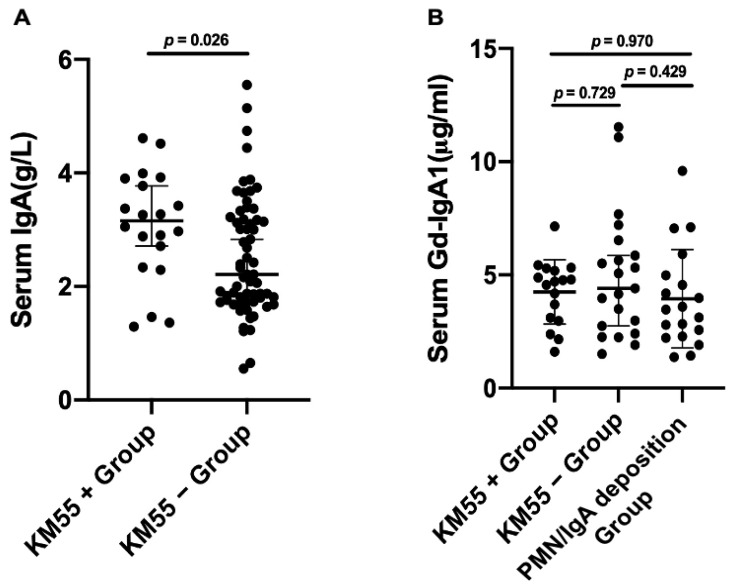
Comparison of the level of serum IgA (**A**) and Gd-IgA1 (**B**) between KM55+ and KM55− subgroup in PMN/IgAN patients. (**A**) The level of serum IgA in KM55+ subgroup was higher than KM55− subgroup in PMN/IgAN patients. (**B**) There was no significant difference in the level of serum Gd-IgA1 in KM55+ subgroup and KM55− subgroup, similar to the PMN/IgA deposition group.

Compared to the KM55− subgroup, the level of serum IgA with KM55+ patients was higher (3.2 versus 2.2 g/L, *p* = 0.026). There was no significant difference in level of serum Gd-IgA1 in KM55+ subgroup, KM55− subgroup, and PMN/IgA deposition group separately (Figure 4).

**Figure 5:**
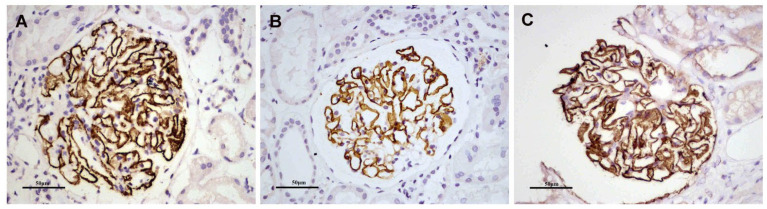
Positive staining for glomerular PLA2R (**A**), NELL-1 (**B**) and THSD7A (**C**) by immunohistochemistry, respectively. (Original magnification ×400).

Because the positive rates of tissue staining for PLA2R were significantly lower in the PMN/IgAN group, we performed other pathogenic antigens (e.g., 7A, NELL-1) for PLA2R-negative patients (*n* = 20) to evaluate PMN antigen difference between KM55+ and KM55− subgroups (Figure 5 and Table 6). Most of the KM55+ patients (85%) were PLA2R associated MN, 10% were NELL-1 associated MN, and only 5% were unknown. The antigen of most of both KM55− and PLA2R- patients was unknown.

### 3.6. Follow up Data between Glomerular KM55+ Subgroup and KM55− Subgroup in PMN/IgAN Patients

**Figure 6:**
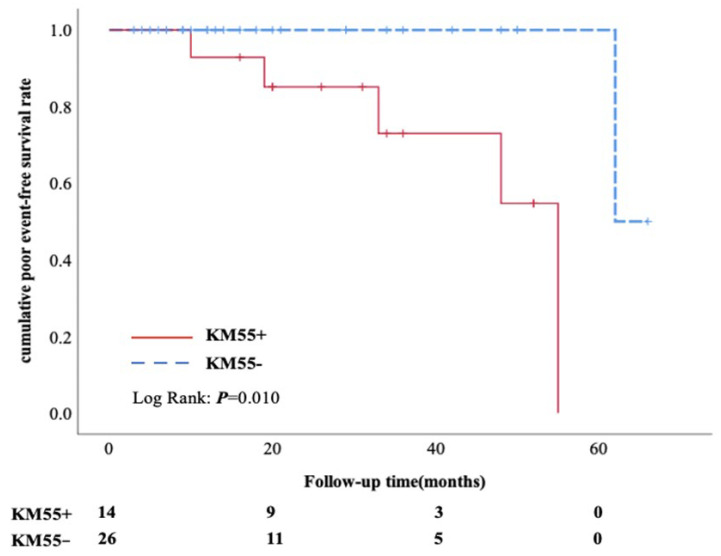
Kaplan-Meier analysis. Cumulative poor event-free renal survival rate between the KM55+ and KM55− subgroups.

Follow-up information was available from 14 cases in KM55+ subgroup and 26 cases in KM55− subgroup. Comparisons between patients with or without complete follow-up data were performed, confirming no selection bias (Supplementary Table S1). There was no significant difference in the percentage of immunosuppressive therapy, including prednisone combined with cyclosporin, cyclosporin only, glucocorticoid only and glucocorticoid combined with cyclophosphamide/mycophenolate mofetil (100% versus 88.5%, *p* = 0.539). 64.3% (9/14) patients in KM55+ subgroup achieved complete or partial remission, which was similar to KM55− group (73.1%, *p* = 0.720) (Table 7). Kaplan-Meier analysis showed that the cumulative incidence of poor events was significantly higher in KM55+ subgroup than in KM55− subgroup(Figure 6). A poor event was defined as an occurrence of a 25% reduction in eGFR or ESRD. Most IgA deposited on glomeruli was not the same pathogenic Gd-IgA1 as in general IgAN.

## 4. Discussion

This study mainly focused on controversial coexisting PMN and IgAN patients. The clinicopathological features of 85 patients with PMN/IgAN were presented. It was noteworthy that disease-specific pathogenic biomarkers and prognosis were explored to better understand the pathological mechanism of these patients.

The overall prevalence of this overlapping disorder was reported as about 1.47–1.75% [7,15]. Because of the low incidence, literature reports only a single case or case series of these PMN/IgAN patents. From the first report by Doi et al. in 1983 [16], an increasing number of studies have reported investigations of the pathogenic mechanism of this coexistent disorder. There has been much controversy. Previous literature has reported that PMN and IgAN may occur in the same patient at an interval of several years [17,18] or on the occurrence of superimposed MN on a background of preexisting IgAN [7,19]. Conclusions are unclear. Recently, there was a larger cohort study including 137 Chinese concurrent IgAN and MN patients, demonstrating that concurrent IgAN and MN may lean towards MN, according to clinicopathological and genetic suggestions [15].

Based on the above research, we speculated that MN may play the main role in this coexisting disease, and coexisting PMN and IgAN may be a special type of PMN. Our results also showed that patients with PMN/IgAN displayed similar clinical and pathological features as PMN/IgA deposition patients. However, it is worth mentioning that lower frequency of nephrotic syndrome, higher serum albumin and a similarly heavy proteinuria were observed in PMN/IgAN patients, indicating that this special coexisting disorder may have some characteristics of IgAN in the context of PMN; similar observations have also been published in other studies [15].

Previous studies mainly focused on clinical and pathological manifestations, but the pathogenesis and prognosis of these coexisting diseases were not well known. Increasing evidence has implied that Gd-IgA1 plays a key role in the pathogenesis of IgAN [20]. In patients with IgAN, Gd-IgA1 has been commonly detected in the renal biopsy specimens, blood and urine. Staining for KM55 in glomeruli of patients with IgAN was relatively specific and displayed excellent diagnostic performance for IgAN [21]. Several studies have suggested that circulating high levels of Gd-IgA1 or urinary Gd-IgA1 may be associated with the development and progression of IgAN [22,23,24,25]. Based on the key role of KM55 in general IgAN, KM55 in circulation and renal tissues were investigated in these PMN/IgAN patients. In our study, the intensity of glomerular KM55 staining in PMN/IgAN patients was 0–3+. Glomerular KM55 positive staining was observed in only 23.5% of PMN/IgAN patients, while no significant difference of serum level of Gd-IgA1 was observed in subgroups according to glomerular KM55 staining, similar to the PMN/IgA deposition patients in our study. Meanwhile, glomerular KM55 staining was all negative in PMN/IgA deposition; this result may indicate no abnormalities in the glycosylation of IgA1, which might provide evidence for excluding IgAN. 

Despite that increasing evidence has implied that KM55 was associated with activity assessment of IgAN [26], there is less evidence concerning KM55 involved in the prognosis of these PMN/IgAN patients. To the best of our knowledge, this is the first study to provide evidence for prognosis in coexisting PMN/IgAN based on investigation of KM55, especially in glomerular KM55 staining. In our study, on the basis of the same percentage of glucocorticoids and other immunosuppressive agents between KM55+ and KM55− subgroup, no significant difference in the rate of partial or complete remission were shown, consistent with He et al. [15]. We speculated that most of our patients had prominent clinical findings of MN that might have led to their hospital presentation; an aggressive treatment regimen was administered according to the guidelines recommended. In addition, we demonstrated that glomerular KM55 positive may predict a poorer prognosis in traditionally diagnosed PMN/IgAN patients by Kaplan-Meier analysis. We assumed that KM55 may play an aggravating role in the background of PMN, but the pathogenesis of these coexisting disorder remains unknown. Further validation from studies of a large sample size and deeper investigation are needed.

When glomerular KM55 staining was positive, this coexisting PMN/IgAN disorder was prone to have more characteristics of IgAN along with PMN, especially affecting the prognosis. However, it is unclear whether the coexisting diseases affect the pathogenetic antigen types of PMN. It is now well known that the target antigen has been identified as PLA2R and 7A in about 70% and 1–5% of PMN [27]. Our previous study reported that one third of patients who were PLA2R and THSD7A negative were NELL-1 positive [14]. In our study, patients with PMN/IgAN had a lower frequency of serum anti-PLA2R antibody and glomerular PLA2R positivity, consistent with other research findings [7,15]. Despite that the positive rates of glomerular PLA2R were lower in the PMN /IgAN group, glomerular IgG4 positive rates were similar. We speculate that this may indicate a higher frequency of other types of target antigens of PMN, such as THSD7A or NELL-1 in PLA2R-negtive PMN/IgAN patients. We further analyzed 20 PLA2R-negtive PMN/IgAN patients. We found that NELL-1 positive accounted for 15% and THSD7A positive accounted for 5%. The antigen of NELL-1 seemed more likely to be positive in the KM55 positive and PLA2R negative patients, but the is just a singular phenomenon, and more samples are needed to verify. Significantly, when glomerular KM55 staining was negative in PLA2R negative PMN/IgAN patients, especially when ANA was positive, secondary factors of MN need to be investigated or closely monitored during follow-up. Exostosin1/ Exostosin2, as helpful biomarkers in lupus nephritis [28,29] should be further studied in these KM55 negative and PLA2R negative PMN/IgAN patients.

## 5. Conclusions

The major limitation of this study is the small sample population and only less than half of the enrolled patients had been regularly followed-up, so progression of renal function should be further validated in long-term follow-up. In addition, there is only an initial exploration of pathogenesis, and deeper investigation is needed.

In conclusion, our study suggested that when glomerular KM55 staining was positive, this special coexisting PMN/IgAN disorder was prone to have more characteristics of IgAN besides PMN, and may predict poorer prognosis, while the mechanism requires further investigation. Our findings may be of importance for judgement and prognosis for this special type of PMN (coexisting PMN and IgAN).

## Figures and Tables

**Table 1:** Demographics and clinical features of patients with PMN/IgAN and PMN/IgA deposition.

Characteristics	PMN/IgAN (*n* = 85)	PMN/IgA Deposition (*n* = 132)	*p* Value
male *n* (%)	58 (68.2)	85 (64.4)	0.560
Age (year)	46.3 ± 14.7	48.5 ± 12.9	0.267
duration of illness (month)	3.0 (1.0, 10.5)	3.0 (1.0, 8.0)	0.766
hypertension *n*(%)	33 (38.8)	61 (46.6)	0.262
microscopic hematuria *n*(%)	44 (51.8)	64 (48.9)	0.676
Proteinuria (g/24h)	5.0 (2.9, 8.8)	5.2 (3.5, 8.0)	0.547
serum albumin (g/L)	27.4 (22.4, 33.5)	25.9 (21.2, 29.6)	0.049
nephrotic syndrome *n* (%)	53 (62.4)	102 (77.3)	0.018
serum creatinine (μmol/L)	70.4 (54.6, 84.4)	66.0 (56.0, 79.2)	0.297
eGFR-EPI (ml/min/1.73 m^2^)	104.0 (89.6, 116.7)	102.6 (89.9, 114.6)	0.901
triglycerides (mmol/L)	2.1 (1.4,2.8)	2.1 (1.4, 3.4)	0.296
total cholesterol (mmol/L)	6.7 (5.3, 7.9)	7.0 (5.7, 9.0)	0.071
LDL-C (mmol/L)	4.1 (2.9, 5.1)	4.4 (3.3, 6.1)	0.091
uric acid (μmol/L)	378.7 ± 85.9	358.9 ± 84.6	0.104
serum IgA (g/L)	2.5 (1.8, 3.4)	2.1 (1.6, 2.9)	0.015
serum IgG (g/L)	6.7 (5.0, 9.2)	5.7 (3.9, 7.4)	0.004
serum IgM (g/L)	0.9 (0.6, 1.3)	1.0 (0.7, 1.3)	0.110
serum C3 (g/L)	1.2 ± 0.3	1.2 ± 0.2	0.094
serum C4 (g/L)	0.3 (0.2, 0.4)	0.3 (0.2, 0.4)	0.952

PMN, primary membrane nephropathy; IgAN, IgA nephropathy; eGFR, estimated glomerular filtration rate; LDL-C, low-density lipoprotein cholesterol; C3, complement C3; C4, complement C4.

**Table 2:** Pathological features of patients with PMN/IgAN and PMN/IgA deposition.

Pathological Features	PMN/IgAN (*n* = 85)	PMN/IgA Deposition (*n* = 132)	*p* Value
Churg stage of MN			0.019
stage I *n* (%)	31 (36.5)	33 (25.0)	
stage II *n* (%)	37 (43.5)	83 (62.9)	
stage III *n* (%)	16 (18.8)	16 (12.1)	
stage IV *n* (%)	1 (1.2)	0 (0)	
glomerular IgG positive *n* (%)	85 (100)	132 (100)	-
glomerular IgG1 positive *n* (%)	34 (40.5)	66 (50.4)	0.155
glomerular IgG2 positive *n* (%)	9 (10.7)	12 (9.2)	0.708
glomerular IgG3 positive *n* (%)	18 (21.4)	18 (13.7)	0.141
glomerular IgG4 positive *n* (%)	76 (90.5)	127 (96.9)	0.065
glomerular IgG4 predominant positive *n* (%)	72 (85.7)	122 (93.1)	0.074
glomerular IgM positive *n* (%)	25 (29.4)	20 (15.2)	0.011
glomerular C1q positive *n* (%)	7 (8.2)	4 (3.0)	0.115
glomerular C3 positive *n* (%)	74 (87.1)	106 (80.3)	0.196
interstitial fibrosis,%, *n* (%)			0.678
<25	73 (85.9)	117 (88.6)	
25–50	10 (11.8)	14 (10.6)	
50–75	2 (2.4)	1 (0.8)	
>75	0 (0)	0 (0)	

PMN, primary membrane nephropathy; IgAN, IgA nephropathy; Immunofluorescence values 2+ and above are positive.

**Table 3:** Disease-specific pathogenic biomarkers for patients with PMN/IgAN.

Characteristics	PMN/IgAN (*n* = 85)	PMN/IgA Deposition (*n* = 132)	*p* Value
serum anti-PLA2R (RU/mL)	8.2 (0.0, 46.1)	47.1 (6.6, 118.3)	<0.001
positive rates of anti-PLA2R *n* (%)	37 (43.5)	81 (61.4)	0.010
glomerular PLA2R positive *n* (%)	65 (76.5)	130 (98.5)	<0.001
glomerular KM55 staining			
mesangial KM55 positive *n* (%)	20 (23.5)	0/29 (0)	0.004
1+	8 (9.4)	0 (0)	
2+	10 (11.8)	0 (0)	
3+	2 (2.4)	0 (0)	
mesangial KM55 negative *n* (%)	65 (76.5)	29/29 (100)	

PMN, primary membrane nephropathy; IgAN, IgA nephropathy; PLA2R, M-type Phospholipase A2 Receptor.

**Table 4:** Demographics and clinical features in PMN/IgAN patients based on the staining of glomerular KM55.

Characteristics	KM55+ (*n* = 20)	KM55− (*n* = 65)	*p* Value
male *n* (%)	14 (70.0)	44 (67.7)	0.846
Age (year)	47.3 ± 13.2	46.0 ± 15.2	0.722
duration of illness (month)	3.5 (1.0, 21.8)	2.0 (1.0, 7.0)	0.138
hypertension *n* (%)	12 (60.0)	21 (32.3)	0.026
microscopic hematuria *n* (%)	9 (45.0)	35 (53.8)	0.489
Proteinuria (g/24h)	3.8 (2.2, 6.3)	5.8 (3.0, 9.3)	0.040
serum albumin (g/L)	28.5 (23.0, 33.1)	27.3 (22.3, 34.2)	0.562
nephrotic syndrome *n* (%)	12 (60.0)	41 (63.1)	0.708
serum creatinine (μmol/L)	75.0 (58.8, 85.2)	68.8 (54.0, 80.2)	0.242
eGFR-EPI (ml/min/1.73 m^2^)	99.9 (81.8, 112.1)	104.4 (91.4, 118.1)	0.325
triglycerides (mmol/L)	2.2 (1.5, 3.7)	2.0 (1.3, 2.7)	0.357
total cholesterol (mmol/L)	6.6 (5.5, 7.1)	6.7 (5.1, 8.0)	0.713
LDL-C (mmol/L)	3.5 (3.0, 4.4)	4.4 (2.9, 5.6)	0.168
uric acid (μmol/L)	389.7 ± 77.0	375.1 ± 88.9	0.510
serum IgA (g/L)	3.2 (2.4, 3.9)	2.2 (1.7, 3.2)	0.026
serum IgG (g/L)	7.1 (4.6, 8.1)	6.5 (5.0, 9.7)	0.782
serum IgM (g/L)	0.8 ± 0.5	1.0 ± 0.4	0.192
serum C3 (g/L)	1.2 ± 0.2	1.2 ± 0.3	0.719
serum C4 (g/L)	0.3 (0.3, 0.4)	0.3 (0.2, 0.3)	0.095

PMN, primary membrane nephropathy; IgAN, IgA nephropathy; eGFR, estimated glomerular filtration rate; LDL-C, low-density lipoprotein cholesterol; C3, complement C3; C4, complement C4.

**Table 5:** Pathological features in PMN/IgAN patients based on the staining of glomerular KM55.

Pathological Features	KM55+ (*n* = 20)	KM55− (*n* = 65)	*p* Value
Churg stage of MN			0.017
stage I *n* (%)	2 (10.0)	29 (44.6)	
stage II *n* (%)	13 (65.0)	24 (36.9)	
stage III *n* (%)	5 (25.0)	11 (12.2)	
stage IV *n* (%)	0 (0)	1 (0.8)	
glomerular IgG positive *n* (%)	20 (100)	65 (100)	-
glomerular IgG1 positive *n* (%)	5 (25.0)	29/64 (45.3)	0.106
glomerular IgG2 positive *n* (%)	1 (5.0)	8/64 (12.5)	0.679
glomerular IgG3 positive *n* (%)	5 (25.0)	13/64 (20.3)	0.756
glomerular IgG4 positive *n* (%)	18 (90.0)	58/64 (90.6)	1.000
glomerular IgG4 predominant positive *n* (%)	18 (90.0)	54/64 (84.4)	0.722
glomerular IgM positive *n* (%)	5 (25.0)	20 (30.8)	0.620
glomerular C1q positive *n* (%)	0 (0)	7 (10.8)	0.191
glomerular C3 positive *n* (%)	17 (85.0)	57 (87.7)	0.715
interstitial fibrosis,%, *n* (%)			0.009
<25	18 (90.0)	55 (84.6)	
25–50	0 (0)	10 (15.4)	
50–75	2 (10.0)	0 (0)	
>75	0 (0)	0 (0)	

MN, membrane nephropathy; C1q, complement C1q; C3, complement C3; Immunofluorescence values 2+ and above are positive.

**Table 6:** Disease-specific pathogenic biomarkers in PMN/IgAN patients based on the staining of glomerular KM55.

Characteristics	KM55+ (*n* = 20)	KM55− (*n* = 65)	*p* Value
serum anti-PLA2R (RU/mL)	15.3 (0.5, 76.3)	5.9 (0, 39.4)	0.241
positive rates of anti-PLA2R *n* (%)	9 (45.0)	28 (43.1)	0.879
glomerular PLA2R positive *n* (%)	17 (85.0)	48 (73.8)	0.379
glomerular PLA2R negative *n* (%)	3 (15.0)	17 (26.2)	
glomerular 7A positive *n* (%)	0(0)	1 (5.9)	
glomerular NELL-1 positive *n* (%)	2 (66.7)	1 (5.9)	
serum Gd-IgA1 (ug/mL) *	4.3 ± 1.4	4.9 ± 2.8	0.332

PLA2R, M-type Phospholipase A2 Receptor; 7A, Thrombospondin Type 1 Domain-Containing 7A; NELL-1, Neural Epidermal Growth Factor–Like 1; Gd-IgA1, galactose-deficient IgA1; *, serums from 17 KM55+ patients and 21 KM55− patients were tested.

**Table 7:** Follow-up data in PMN/IgAN patients based on the staining of glomerular KM55.

Characteristics	KM55+ (*n* = 14)	KM55− (*n* = 26)	*p* Value
remission *n* (%)	9 (64.3)	19 (73.1)	0.720
complete remission *n* (%)	4 (28.6)	6 (23.1)	0.718
partial remission *n* (%)	5 (35.7)	13 (50.0)	0.386
immunosuppressive therapy *n* (%)	14 (100.0)	23 (88.5)	0.539
prednisone and cyclosporin			
remission *n* (%)	2/3 (66.7)	3/5 (60.0)	1.000
cyclosporin			
remission *n* (%)	5/7 (71.4)	9/12 (75.0)	1.000
other			
remission *n* (%)	2/4 (50.0)	5/6 (83.3)	0.500
composite outcome *n* (%)	5 (35.7)	1 (3.8)	0.014

Other treatment plans, such as glucocorticoid and glucocorticoid combined with cyclophosphamide/ mycophenolate mofetil; composite outcome was defined as a 25% decline in eGFR or ESRD.

## Data Availability

The data presented in this study are available on request from the corresponding author.

## Supplementary Materials

The following are available online at https://www.mdpi.com/article/10.3390/cells12010116/s1, Table S1. The main baseline clinicopathological features of patients regularly followed-up and Lost follow-up patients, Table S2: The main baseline clinicopathological features in PMN/IgAN patients regularly followed-up based on the staining of glomerular KM55.
