# Supplementary material for: Glomerular Galactose-Deficient IgA1(KM55) Positive May Predict Poorer Prognosis in Coexisting Primary Membranous Nephropathy and IgA Nephropathy Patients
Source: Cells. 2022 Dec 28;12(1):116. doi: 10.3390/cells12010116 (PMC9818871; doi:10.3390/cells12010116)
Supplement: Supplementary file 1 [file cells-12-00116-s001.zip › cells-2009822-supplementary.pdf]

Supplementary Table 1 The main baseline clinicopathological features of patients regularly followed-up and Lost follow-up patients

Characteristics	Group A (patients followed-up) （n=40）	Group B (patients lost follow-up) （n=45）	P value
male n(%)	26(65.0)	32(71.1)	0.546
age(year)	48.1±13.4	44.6±15.7	0.281
microscopic hematuria n(%)	20(50.0)	24(53.3)	0.759
proteinuria(g/24h)	5.2(3.2,9.3)	4.7(2.6,8.3)	0.359
serum albumin (g/L)	27.9(22.4,34.1)	27.3(22.3,33.1)	0.819
nephrotic syndrome n(%)	26(65.0)	28(62.2)	0.791
serum creatinine(μmol/L)	71.3(54.5,81.6)	66.0(55.6,86.0)	0.996
eGFR-EPI(ml/min/1.73 m^2)	104.5(92.1,114.8)	100.8(90.6,119.0)	0.944
uric acid(μmol/L)	372.0±81.3	385.0±90.5	0.498
serum IgA(g/L)	2.8(1.9,3.5)	2.3(1.7,3.4)	0.220
serum IgG(g/L)	6.6(4.5,10.3)	6.8(5.0,9.0)	0.795
serum anti-PLA2R(RU/ml)	8.5(0.0,76.3)	8.2(0.0,35.5)	0.431
positive rates of anti-PLA2R n(%)	17(42.5)	20(44.4)	0.857
glomerular PLA2R positive n(%)	30(75.0)	35(77.8)	0.763
interstitial fibrosis,%, n(%)			1.000
<25	34(85.0)	39(86.7)	
25-50	5(12.5)	5(11.1)	
50-75	1(2.5)	1(2.2)	
＞75	0(0)	0(0)	

eGFR, estimated glomerular filtration rate; PLA2R, M-type Phospholipase A2 Receptor

Supplementary Table 2 The main baseline clinicopathological features in PMN/IgAN patients regularly followed-up based on the staining of glomerular KM55

Characteristics	KM55+ (patients followed-up) （n=14）	KM55- (patients followed-up) （n=26）	P value
male n(%)	8(57.1)	18(69.2)	0.501
age(year)	48.5±12.4	47.9±14.2	0.892
proteinuria(g/24h)	4.4±2.5	7.8±5.8	0.013
serum albumin (g/L)	29.3±7.4	28.1±7.9	0.635
nephrotic syndrome n(%)	9(64.3)	17(65.4)	1.000
serum creatinine(μmol/L)	77.2(56.0,83.5)	69.6(54.3,78.4)	0.234
eGFR-EPI(ml/min/1.73 m^2)	99.9(77.1,110.5)	105.8(89.7,116.3)	0.223
serum anti-PLA2R(RU/ml)	38.9(1.6,137.5)	4.6(0.0,48.6)	0.202
positive rates of anti-PLA2R n(%)	7(50.0)	10(38.5)	0.481

glomerular PLA2R positive n(%)	11(78.6)	19(73.1)	1.000
interstitial fibrosis,%, n(%)			0.083
<25	13(92.9)	21(80.8)	
25-50	0(0)	5(19.2)	
50-75	1(7.1)	0(0)	
＞75	0(0)	0(0)	

eGFR, estimated glomerular filtration rate; PLA2R, M-type Phospholipase A2 Receptor
